# Optogenetic Neuronal Stimulation Promotes Functional Recovery After Spinal Cord Injury

**DOI:** 10.3389/fnins.2021.640255

**Published:** 2021-04-09

**Authors:** Wei-wei Deng, Guang-yan Wu, Ling-xia Min, Zhou Feng, Hui Chen, Ming-liang Tan, Jian-feng Sui, Hong-liang Liu, Jing-ming Hou

**Affiliations:** ^1^Department of Rehabilitation, Southwest Hospital, Army Medical University, Chongqing, China; ^2^Experimental Center of Basic Medicine, College of Basic Medical Sciences, Army Medical University, Chongqing, China

**Keywords:** spinal cord injury, motor cortex, functional recovery, optogenetics, glutaminergic neurons

## Abstract

Although spinal cord injury (SCI) is the main cause of disability worldwide, there is still no definite and effective treatment method for this condition. Our previous clinical trials confirmed that the increased excitability of the motor cortex was related to the functional prognosis of patients with SCI. However, it remains unclear which cell types in the motor cortex lead to the later functional recovery. Herein, we applied optogenetic technology to selectively activate glutamate neurons in the primary motor cortex and explore whether activation of glutamate neurons in the primary motor cortex can promote functional recovery after SCI in rats and the preliminary neural mechanisms involved. Our results showed that the activation of glutamate neurons in the motor cortex could significantly improve the motor function scores in rats, effectively shorten the incubation period of motor evoked potentials and increase motor potentials’ amplitude. In addition, hematoxylin-eosin staining and nerve fiber staining at the injured site showed that accurate activation of the primary motor cortex could effectively promote tissue recovery and neurofilament growth (GAP-43, NF) at the injured site of the spinal cord, while the content of some growth-related proteins (BDNF, NGF) at the injured site increased. These results suggested that selective activation of glutamate neurons in the primary motor cortex can promote functional recovery after SCI and may be of great significance for understanding the neural cell mechanism underlying functional recovery induced by motor cortex stimulation.

## Introduction

Spinal cord injury (SCI) is a serious trauma of the central nervous system and one of the main causes of human disability (Harvey et al., 1992; Cadotte and Fehlings, 2011). SCI often leads to limb motor dysfunction, which in turn may lower the patient’s quality of life, cause the loss of labor, and bring a huge burden to society and families (Ahmad et al., 2015). Restoring extremity functions of patients with SCI can make many activities of daily living reality, which may significantly decrease the social and economic burdens. Consequently, researchers have carried out a lot of research on nerve repair after SCI in the past decades. Over recent years, great progress has been made in understanding the mechanism of secondary injury of SCI. Several treatments have been proposed, including cell transplantation (Kojima et al., 2019), the application of neurotrophic drugs (Fournier et al., 2003; Sung et al., 2003; Kitzman, 2009), and tissue engineering technology. However, these treatments still lack effective intervention in clinic (Onose et al., 2009).

Previous studies mainly focused on the local area of SCI while ignoring the brain that is closely related to the spinal cord’s structure and function. At present, most of the studies on the repair of SCI are based on the fact that the brain structure and function remain normal after SCI. However, the brain’s structure and function after SCI have been confirmed to change over recent years and may have a significant impact on the functional recovery of SCI.

Over the past years, we have carried out a series of studies on exploring the changes in brain structure and function after SCI. We identified structural and functional remodeling of the cerebral motor-related cortex (primary motor cortex, auxiliary motor area, and premotor area, etc.) in patients with SCI, which can occur in the early stage of injury (Hou et al.,2014a,b). Our follow-up study confirmed that the correlations between spontaneous nerve activity in the primary motor cortex (M1 area) of the brain and the functional recovery that is the higher the excitability of the M1 area after SCI, the better the recovery of motor function after 6 months (Hou et al., 2016). A previous studies showed that the increase of neuronal excitability in the brain’s M1 area after SCI is the main reason for functional recovery. When neurons’ activity in the M1 area was inhibited, the recovery of finger flexibility in rhesus monkeys was significantly affected (Nishimura et al., 2007). The above results suggest that the M1 region has a very important role in late functional recovery after SCI. Several methods, including transcranial magnetic stimulation (TMS) and electrical stimulation, can improve the excitability of the motor cortex in the treatment of SCI (Tazoe and Perez, 2015). Studies have also confirmed that the application of TMS (especially high-frequency magnetic stimulation can improve neuronal excitability) or electrical stimulation could promote the recovery of motor function in patients with SCI (Hirschfeld et al., 2008; Creasey and Craggs, 2012; Wein, 2013). However, although the above neural regulation methods have some clinical effects, these are not as significant as expected. We speculate that although electrical or magnetic stimulation activates excitatory neurons (glutamatergic neurons) in the M1 area, it activates inhibitory neurons (GABA neurons) in the M1 area, and the range of activation cannot be accurately limited. Therefore, it may simultaneously lead to the activation of excitatory and inhibitory neurons in the M1 area, finally affecting the clinical results.

Optogenetics is a rapidly developing bioengineering technology that integrates optics, software control, gene manipulation, electrophysiology, and other disciplines (Ahmad et al., 2015). This technique has the advantages of high cell-type specificity, non-invasive, high spatial resolution, accurate location, and repeatability. Based above, we propose that the precise activation of M1 excitatory glutamatergic neurons but not the activation of GABAergic inhibitory neurons, which is may be play a role in promoting the recovery of motor function after SCI. Optogenetic technology has been widely used in various neuropsychiatric diseases, especially in treating epilepsy (Paz and Huguenard, 2015), mental illness (Deisseroth, 2012), dyskinesia (Rossi et al., 2015), and central pain (Lee and Kim, 2016). As we know, few research studied on the use of optogenetics activated M1 glutaminergic neurons to promote functional recovery after SCI.

In this study, we used an optogenetic technique to selectively activate pyramidal excitatory neurons in the M1 area so as to avoid the activation of inhibitory neurons in the M1 area and to observe the recovery of motor function after SCI. Furthermore, we also analyzed the expression of neurotrophic factors in SCI to explore the neural mechanism underlying the excitability of M1 excitatory neurons in the recovery of motor function in the later stage.

## Materials and Methods

### Experimental Animals and Grouping

All animals and surgical procedures were performed in accordance with the guidelines approved by the animal ethics committee of the first affiliated hospital of Army Medical University (Chongqing, China). 90 adult female Sprague-Dawley rats (weight, 300 *g*) were purchased from the animal center of Army Medical University (Chongqing, China). Animals were housed (two rats per cage) in standard laboratory cages using a 12/12-h light/dark cycles at 24 ± 2°C, with *ad libitum* access to food and water throughout the experimental procedures.

**FIGURE 1:**
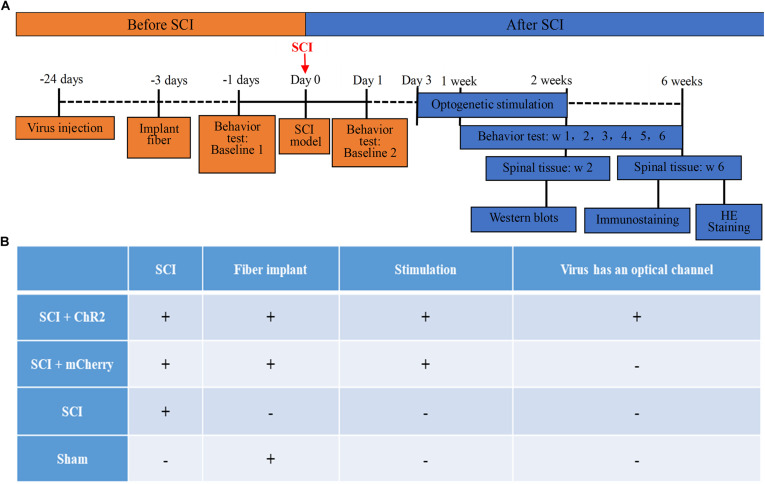
The timeline and grouping diagram of this study. **(A)** The overall timeline of the experiment. virus injection and installation of optical fiber porcelain head need to be completed about 3 weeks before SCI modeling. Light stimulation is carried out from 3 days to 2 weeks after successful modeling. Spinal cord tissue samples are taken after stimulation, which are used for WB detection of protein. The weekly behavioral score starts from the first week after modeling and ends at the sixth week. At the 6th week, spinal cord tissue was collected for immunofluorescence staining and HE staining of nerve fibers at spinal cord injury. **(B)** The grouping of this study and the corresponding treatment methods of each group. Animals were divided into four groups: SCI + ChR2 group (Spinal cord injury + Injected with ChR2 + Implanted with the cannula delivering blue light into M1 area), SCI + mCherry group (Spinal cord injury + Injected with mCherry + Implanted with the cannula delivering blue light into M1 area), SCI group (Only spinal cord injury), and Sham group (Sham spinal cord injury + Implanted with the cannula delivering blue light into M1 area).

The animals were randomly divided into four groups: SCI + ChR2 (SCI + Injected with ChR2 + Implanted with the cannula delivering blue light into M1 area, *n* = 6), SCI + mCherry (SCI + Injected with mCherry + Implanted with the cannula delivering blue light into M1 area, *n* = 6), SCI (Only SCI, *n* = 6), and Sham (Sham SCI + Implanted with the cannula delivering blue light into M1 area, *n* = 6). See Figure 1 for more details about overall arrangement of the experiment and treatment of each group.

### Virus Injection

The injection method and dose of the virus referred to our previous studies (Su et al., 2019). Briefly, rats were first subjected to anesthesia (3% pentobarbital sodium, 40 mg/kg, i.p.) and then anchored in a digital stereotaxic frame (Model 942, David Kopf Instruments, United States). Then, an incision was made on the top of the scalp, and two small holes were drilled on each side of the skull centered on the bilaterally M1 area. For optogenetic selectively activate pyramidal neurons in the M1 area, we slowly injected 0.3 μl of rAAV 2/9-CaMKIIα- ChR2(H134R)-mCherry (Virus titers: 1.35 × 1013 GC/mL) or rAAV 2/9-CaMKIIα-mCherry (Virus titers: 1.25 × 1013 GC/mL) as a control obtained and packaged by Taitool Bioscience (Shanghai, China) into bilaterally M1 area [anteroposterior (AP) + 0.00 mm from bregma, mediolateral (ML) ± 1.9 mm, and dorsoventral (DV) – 2.0 mm] with a microinjector at the rate of 0.05 μl/min controlled by a stereotaxic microsyringe pump. Rats in the sham group will not inject any drugs in M1 area except for embedding optical fiber porcelain head. After injection, the needle was left in place for 5 min before withdrawal to allow the virus to diffuse into the surrounding tissue. After all of that, rats were monitored for recovery and were returned to home cages.

### Optical Fiber Implantation and Blue Laser Light Stimulation

The optical fiber implantation methods and laser light stimulation parameters referred to our previous studies and other related articles (Su et al., 2019). The specific steps were as follows: optical fibers were implanted at about 4 weeks after virus injection, rats were anesthetized with 3% pentobarbital sodium (40 mg/kg, i.p.) and mounted on a digital stereotaxic frame (Model 942). Briefly, for optogenetic manipulations, two optical fiber cannulae (ceramic ferrule: diameter 2.50 mm; optical fiber: 200 μm core diameter, 0.37 NA, inper, China) were implanted 300 μm above the viral injection site into the bilateral M1 area (AP + 0.00 mm, ML ± 2.00 mm, and DV – 1.5 mm). Ultimately, the optical fiber cannulae were cemented to the skull with dental cement. Blue laser illumination was presented to activate the pyramidal neurons in the M1 area and controlled by a pulse stimulator (Master-9, A.M.P.I.). The power intensity of laser illumination at each fiber tip in brain tissue was measured with a digital optical power and energy meter (PM100D, Thorlabs). For *in vivo* optogenetic activation, rats received 12-min stimulation–rest cycles illumination (1 min laser on–3 min laser off, repeated 3 times) with a 473-nm laser (∼5 mW, 20 Hz, and 15 ms) controlled by a pulse stimulator (Master-9). The rats received 3 times illumination per day for a total of 2 weeks of light stimulation.

### Establishment of the SCI Model

Three days after optical fibers were implanted, rats were anesthetized with 5% chloral hydrate (7 ml/kg body weight) and placed on a stereotaxic frame to expose the spinal cord at T 8–10 lamina. Each rat’s spinal cord was clearly exposed and performed using a 50 *g* aneurysm clip to compress the T9 spinal cord for 60 s. Tail shaking and hindlimb reflexes were used as indicators of the successfully established SCI model (Choi et al., 2012). Rats in the sham group only needed to cut the skin of T8–T10 and remove the corresponding bone. After the operation, rats were subcutaneously injected with penicillin (200,000 U/animal/day) before being placed back for 7 consecutive days following the operation so as to prevent infection. General health, infections, and mobility of the rats were monitored twice every day throughout the post-injury survival period. Postoperative care included manual expression of bladders twice a day until the rats reached spontaneous micturition.

### Behavior Tests

The Basso, Beattie, and Bresnahan (BBB; Basso et al., 1995) locomotor rating scale and the inclined plane test (Rivlin and Tator, 1977) were used to assess the recovery of locomotor functions on day 1 and weeks 1, 2, 3, 4, 5, and 6 post-SCI modeling. The above two behavior tests were performed and analyzed by two blind investigators. The higher the BBB score or the slope stability angle, the better the motor function recovery of the rat. The specific steps of the above behavioral tests were as follows: In the BBB score test, we first put the rats to be tested one by one into the iron fence with a radius of about 70∼100 cm, and then hit the iron wall to let the rats crawl in the iron fence for 1–2 min. At the same time, the hip, knee and ankle walking and body movement process and coordination ability of the rats were observed. Finally, the motor function of the rats was evaluated according to the BBB rating scale. In the slope test, the rats were placed one by one on the inclined plate of 20°∼90°, raised 2.5°∼5°each time, and rested 5 min before each angle test. When the rats were stable for 10 s or more at a certain slope and repeated for 3 times, the slope would be recorded as the stable slope of the rats, and the maximum stable slope of the rats would be recorded. The behavioral tests were conducted by two researchers in the laboratory who did not know the grouping of the experiment. All the behaviors were recorded and photographed by mobile phone.

### Motor Evoked Potential Examination

Six weeks after SCI, adult female Sprague-Dawley rats were used for motor evoked potentials (MEP) and anesthetized with a single intraperitoneal (i.p.) injection of sodium pentobarbital at 25 mg/kg, which still made the animals maintain hindpaws withdrawal reflex when stimulating the toes. Immediately, the rats were fixed to the board, and the stimulation needle electrodes were placed 2 mm in front of the crown suture and 2 mm in the midline under the scalp. The recording electrode was placed in the middle of the tibial anterior muscle group, and the ground needle electrode was placed in rat’s tail (Redondo-Castro et al., 2016). The intensity of a single stimulus was 10–25 mV. The latency was in milliseconds (ms) and the amplitude is in microvolts (mV).

### HE Staining (Histology)

For hematoxylin-eosin staining (HE), Spinal cord segments T10 containing the injury region were fixed in 4% paraformaldehyde (PFA, prepared in 0.1 M of phosphate buffer, pH 7.4) at 4°C for 24 h. Following this, the tissue sections were transferred to 75, 85, 95, and 100% different gradients of ethanol for 15 min and carried out with dehydration, respectively. After this, the spinal cord segments T10 were treated with different levels of ethanol, xylene, and paraffin for transparency and paraffin embedding. The next experiment included cutting 4 μm-thick coronal sections on a freezing microtome (CM3050 S, Leica) and after deparaffinization and rehydration, 4 μm longitudinal sections were stained with hematoxylin solution for 5 min followed by 5 dips in 1% acid ethanol (1% HCl in 70% ethanol) and then rinsed in distilled water. Then the sections were stained with eosin solution for 3 min and followed by dehydration with graded alcohol and clearing in xylene. Finally, the extent of the tissue sections of the injury region was carefully checked, and their images were acquired using an Olympus VS200 fluorescence microscope (Japan) using a 20× air objective.

### Protein Extractions and Western Blots

Western blots analysis was performed on day 14 after SCI. Rats were sacrificed and the T10 spinal cord segments (0.5 cm above and below the injured epicenter) were collected after transcardial perfusion with sterile saline. Take the spines of the cut up and transfer to the homogenate, PMSF is added in the precooling of pyrolysis buffer, and the pyrolysis buffer in homogenate device quickly and continue to make full grinding, and grinding fluid is transferred to the group 1.5 ml centrifuge tube, then 12000 RPM centrifugal 5 min under 4°C, take that repackaging supernatant fluid in 0.5 ml centrifuge tube, and then use BCA kit to determine protein concentration. Equal amounts of protein were separated by SDS/PAGE gels, and the proteins were transferred onto PVDF membranes. The blots were blocked with 5% milk for 1 h at room temperature and were subsequently incubated overnight at 4°C with the following primary antibodies: GAP-43 (NOVUS, NB300-143), NGF (abcam,Ab52918), and BDNF (Abcam,Ab198319). Membranes were washed and incubated with corresponding secondary antibodies for 1 h at room temperature. The antibodies were visualized using ECL detection reagent. The relative densities of the bands were normalized to GADPH and analyzed using Image J.

### Statistical Analysis

All of the data were expressed as the mean ± standard error of the mean (s.e.m.). The statistical significance was determined by one-way analysis of variance (ANOVA) followed by Tukey’s *post hoc* test, or by a two-way ANOVA with repeated measures followed by Tukey’s *post hoc* test using the SPSS software for the Windows package (v. 25.0). A value of *P* < 0.05 was considered to be statistically significant.

## Results

### Activation of M1 Glutaminergic Neurons

**FIGURE 2:**
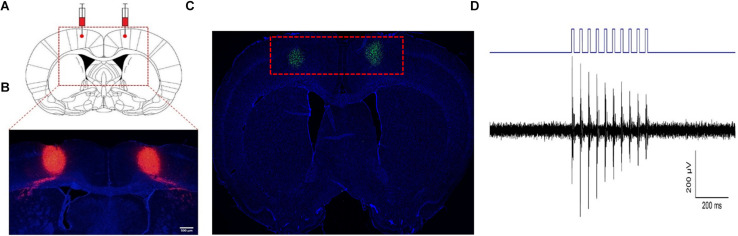
Activation of M1 glutaminergic neurons. **(A, B)** The region where the virus is injected and expressed. **(A)** The red dotted line box is the anatomical diagram of the rat’s brain injected with the virus. **(B)** The red dotted line box is the expression of the virus at the corresponding position. **(C)** c-Fos staining of the M1 region after virus expression showed that c-Fos expression was found in the green part. **(D)** Electrophysiological detection of nerve cell discharge during light stimulation.

Three weeks after the rats were injected with the adeno-associated virus, DAPI staining showed obvious and stable expression of the virus in the bilateral M1 region (Figures 2A,B), while c-Fos staining of the brain also showed activation of M1 glutaminergic neurons (Figure 2C). By installing fiber-optic porcelain heads on rats, 470 nm blue light was used to stimulate the M1 region of the rat brain. The discharge of cells during stimulation was detected, revealing significantly increased discharge of stimulated cells in the M1 region of rats (Figure 2D). The stimulated rats were also observed by a camera. The rats’ hind limbs had strong involuntary movement during light stimulation that stopped when the light stimulation was stopped (Supplementary Movie 1). During the whole process, all physiological characteristics of the rats were normal, and no other mental symptoms such as epilepsy occurred. These results proved that the injected virus could activate M1 glutaminergic neurons and control the movement of rats’ hind limbs through light.

### Accurate Simulation of M1 Glutaminergic Neurons Promotes Functional Recovery After SCI in Rats

**FIGURE 3:**
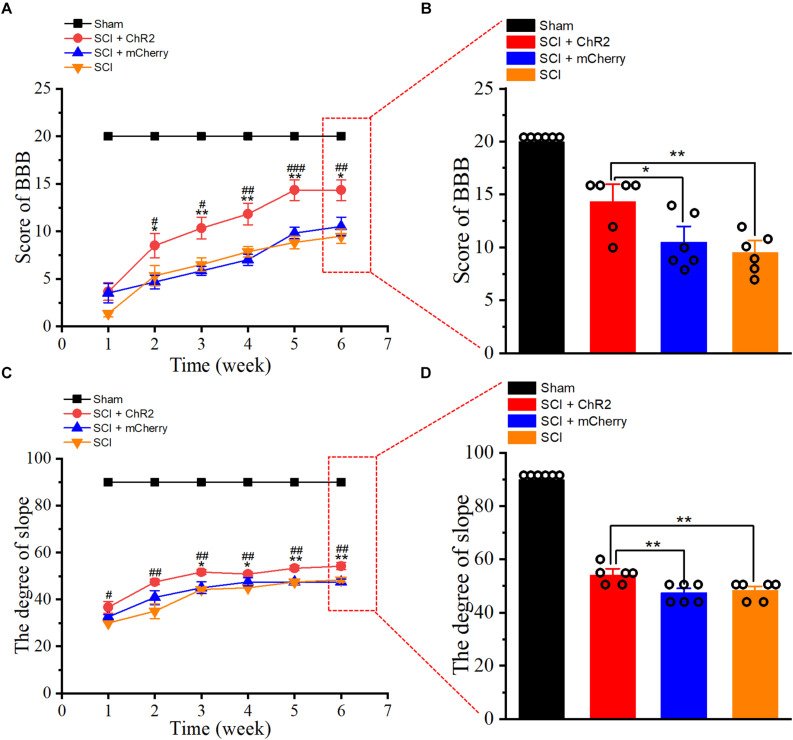
Accurate stimulation of M1 glutamatergic neurons promotes functional recovery after SCI in rats. **(A)** The line chart of BBB score of rats at 1–6 weeks after SCI. **(B)** The histogram of BBB score of rats at 6 weeks after SCI. **(C)** The line diagram of ramp stability angle of rats 1–6 weeks after SCI. **(D)** The ramp stability angle diagram of rats 6 weeks after SCI. [in chart **A, C**: * indicates *P* < 0.05 between SCI + ChR2 group and SCI + mCherry group, **indicates *P* < 0.01, ^#^indicates *P* < 0.05 between SCI + ChR2 group and SCI group, ^##^indicates *P* < 0.01, ^###^indicates *P* < 0.001; in chart **B, D** histograms: * indicates *P* < 0.05, **indicates *P* < 0.01, *n* = 6 rat/group, using one-way ANOVA. Data are shown as means (bar graphs) and standard error of the mean (error bars), with individual data points indicated as circles].

At 1–6 weeks after SCI, we tested the weekly functional changes of SCI rats’ hind limbs through BBB score and slope test. Our results revealed that BBB score (Figure 3A) and slope stability angle (Figure 3C) of injured groups improved with time. Notably, from week 2 to week 6, the weekly scores in the SCI + ChR2 group were higher than those in the SCI group and SCI + mCherry group (*P* ¡ 0.05). By the 6th week of the experiment, the BBB score (Figure 3B) and slope stability angle (Figure 3D) in the SCI + ChR2 group were significantly better compared to the above two SCI groups (*P* ¡ 0.05), and there was no significant difference between the test results of SCI + mCherry and SCI groups (*P* ¿ 0.05). The above findings indicated that light accurate stimulation of M1 glutaminergic neurons could promote the motor function of SCI rats and had obvious advantages in long-term functional recovery.

### Accurate Simulation of M1 Glutamatergic Neurons Can Excite Descending Conduction Bundles After SCI in Rats

**FIGURE 4:**
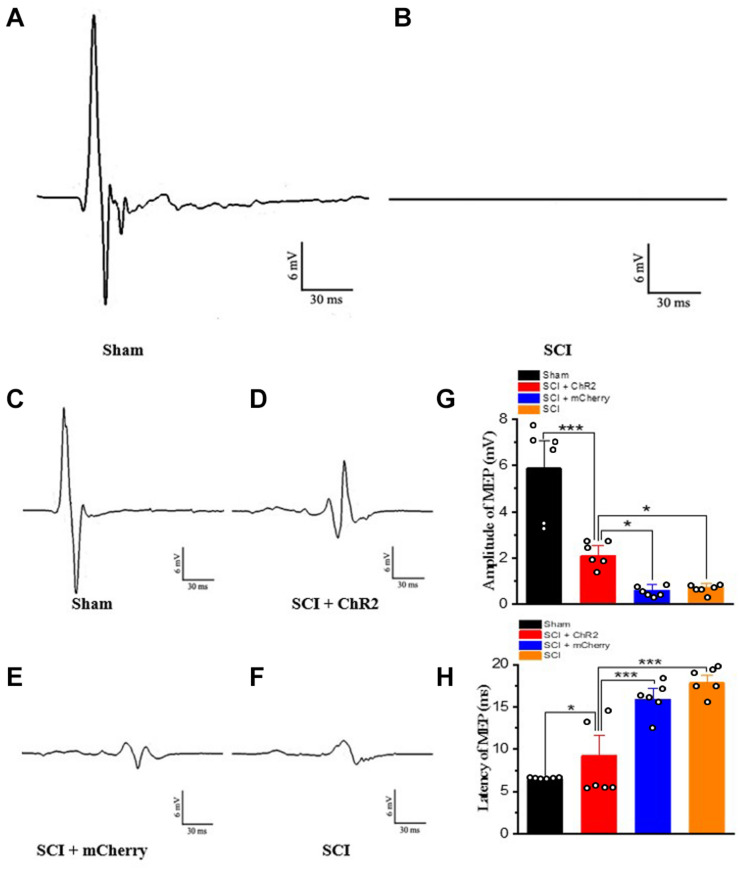
Accurate stimulation of M1 glutamatergic neurons in rats after SCI. **(A)** The results of motor evoked potentials on the first day after operation in the sham group. **(B)** The results of motor evoked potentials of SCI rats on the first day after the operation. **(C–F)** The test results of motor evoked potentials of the corresponding groups at the 6th week, respectively. **(G)** The histogram display of the amplitude of motor evoked potentials of each group. **(H)** The histogram display of the incubation period of the motor evoked potentials of each group. [*indicates *P* < 0.05, *** indicates *P* < 0.001, *n* = 6 rat/group, using one-way ANOVA. Data are shown as means (bar graphs) and standard error of the mean (error bars), with individual data points indicated as circles].

In order to confirm whether light accurate stimulation of the M1 glutaminergic neurons in rats could activate descending conduction bundle, thus triggering hind limb movement in SCI rats, we first detected MEPs on the first day after SCI in rats. Compared to the MEPs of sham group rats (Figure 4A), almost no MEPs were generated in rats after SCI (Figure 4B). At week 6 after SCI, we re-tested the MEPs of rats in each group (Figures 4C–F). The amplitudes of MEPs in SCI + ChR2 group were higher than those of the SCI group and SCI + mCherry group (Figure 4G; *P* ¡ 0.05). Moreover, the incubation period in the SCI + ChR2 group was also significantly better than that of the above two SCI groups (*P* ¡ 0.001, Figure 4H). In addition, there were no significant differences between SCI + mCherry group and SCI group on the test results of MEPs and incubation period (*P* ¿ 0.05). Above all, the above findings indicated that the precise activation of M1 glutamatergic neurons could excite the descending conduction bundle, thus triggering the movement of hind limbs and ultimately promoting the recovery of motor function in SCI rats.

### Accurate Simulation of M1 Glutamatergic Neurons Promotes Tissue Recovery at the Injured Site After SCI in Rats

**FIGURE 5:**
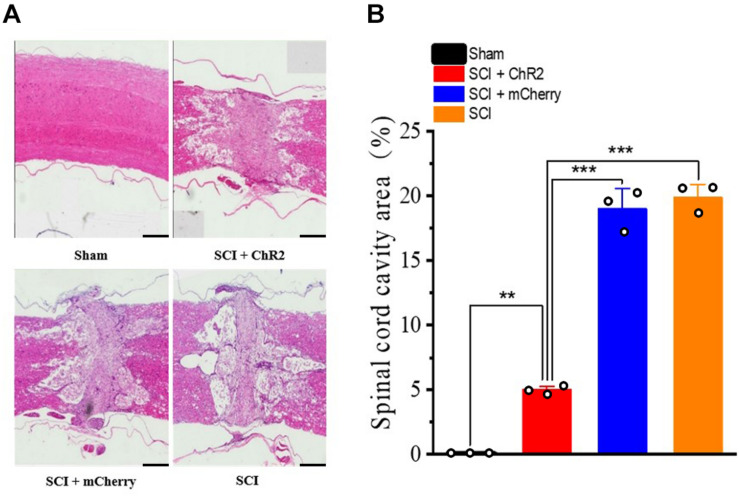
Accurate stimulation of glutamatergic neurons in the M1 region promotes injured tissue recovery after spinal cord injury in rats. **(A)** The comparison chart of HE stained tissue sections of the four groups of rats at the 6th week (500 μm/bar). **(B)** The histogram of the injury area of each group of spinal cord injury. [**indicates *P* < 0.01, ***indicates *P* < 0.001, *n* = 3 rat/group, using one-way ANOVA. Data are shown as means (bar graphs) and standard error of the mean (error bars), with individual data points indicated as circles].

In order to further explore why the functional recovery at the 6th week of SCI rats after accurate simulation of M1 glutamatergic neurons, we used HE staining to observe the histological changes of the four groups. It was found that the tissue in the injured area of the SCI + ChR2 group was clearer, while the cavity area was smaller (Figure 5A). We further calculated the cavity area for statistical analysis and found that the cavity area of the SCI + ChR2 group was significantly smaller than those of the SCI group and SCI + mCherry group (*P* ¡ 0.001, Figure 5B). These findings indicated that accurate activation of M1 glutamatergic neurons could promote the repair of damaged tissues in SCI rats.

### Accurate Simulation of M1 Glutaminergic Neurons Increased the Expression of Neurotrophins After SCI in Rats

**FIGURE 6:**
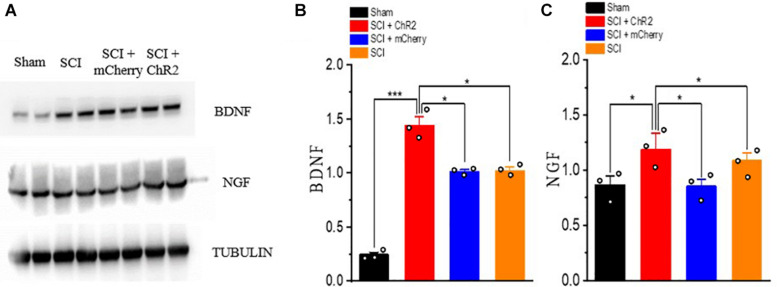
Accurate stimulation of M1 glutamatergic neurons increased the expression of neurotrophin after SCI in rats. **(A)** WB bands of nerve growth-related factor BDNF and NGF proteins. **(B,C)** Expression of BDNF and NGF protein. [protein content = target protein gray value/internal reference gray value; *P < 0.05, ***P < 0.001, *n* = 3 rat/group, using one-way ANOVA. Data are shown as means (bar graphs) and standard error of the mean (error bars), with individual data points indicated as circles].

The expression of neurotrophic factor (BDNF, NGF) in the injured spinal cord was detected by Western blots (Figure 6A). The results showed that the expression levels of BDNF and NGF were higher in each injury group than that in Sham group (*P* ¡ 0.05). Moreover, the expression levels of BDNF and NGF in SCI + ChR2 group was higher than those in SCI + mCherry group and SCI group (*P* ¡ 0.05). The degree of increase of BDNF and NGF in SCI + mCherry group was similar to that in the SCI group (*P* ¿ 0.05, Figures 6B,C). These results showed that the precise activation of the M1 glutaminergic neurons could promote the increase expression of BDNF and NGF in the injured area, thus promoting the repair of the injured tissue and finally promoting the recovery of motor function in SCI rats.

### Accurate Simulation of M1 Glutaminergic Neurons May Promotes Regeneration of Neurofilaments at Injury Site After SCI in Rats

**FIGURE 7:**
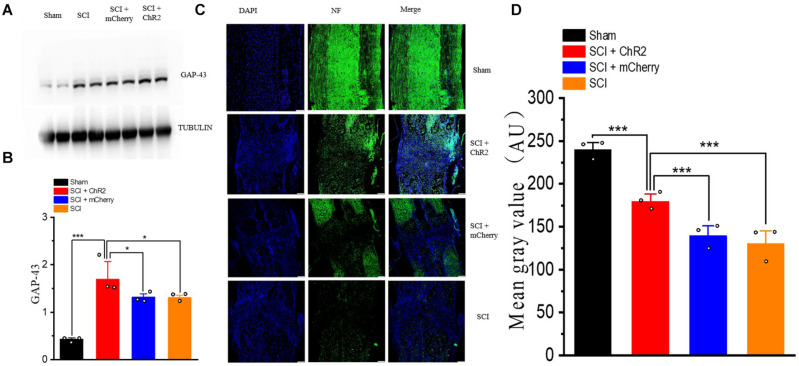
Accurate stimulation of M1 glutamatergic neurons promotes the regeneration of nerve fibers after SCI in rats. **(A)** Display of WB bands of nerve growth-related molecule GAP-43. **(B)** Statistical analysis histogram of GAP-43 gray value. **(C)** Immunofluorescence staining of NF (neurofilament) in spinal cord tissue of rats in each group at the 6th week, blue staining for DAPI, and green staining for NF expression (200 μm/bar). **(D)** Histogram of Mean Gray Value. [Protein content = target protein gray value/internal reference gray value; *P < 0.05, ***P < 0.001, *n* = 3 rat/group, using one-way ANOVA. Data are shown as means (bar graphs) and standard error of the mean (error bars), with individual data points indicated as circles].

We also detected the expression level of nerve growth-related molecules such as GAP-43, which has an important role in axial germination and synaptic plasticity. The results showed that the expression level of GAP-43 increased in all SCI groups, and the expression level of the SCI + ChR2 group was higher than those of the SCI + mCherry group and SCI group (*P* ¡ 0.05, Figures 7A,B). In addition, we used immunofluorescence staining to detect the formation of nerve fibers (Figure 7C) in the injured spinal cord of rats in each group at the 6th week. The results showed that the fluorescence expression of nerve fibers in the injured spinal cord of the SCI + ChR2 group was slightly higher than that of other injured groups (*P* ¡ 0.05, Figure 7D). These findings indicated that accurate simulation of M1 glutaminergic neurons on SCI rats can promote neurofilament regeneration in the injured spinal area, thus promoting repair of injured tissues and ultimately promoting recovery of motor function in SCI rats.

## Discussion

Motor dysfunction is commonly found in patients with SCI, considerably affects the quality of life. The purpose of this study was to investigate whether the specific activation of M1 glutaminergic neurons of rats after SCI can promote the recovery of their motor function. Our results showed that the specific activation of M1 glutaminergic neurons could increase the content of neurotrophic factors in the injured spinal cord, thus promoting the regeneration of nerve fiber axons and improving the supporting and grasping ability of rat hind limbs after SCI. Therefore, these results suggest that accurate stimulation of M1 glutaminergic neurons may be an effective way to treat motor dysfunction after SCI. To the best of our knowledge, this study is the first one that specifically activated M1 glutaminergic neurons through optogenetics technology to promote functional recovery after SCI in rehabilitation field.

One of the most important findings of this study is that stimulation of M1 region of the brain can promote the functional recovery of SCI rats, which points out an effective stimulation target for the current neuromodulation therapies on SCI. Recently, most medical treatments for SCI are aimed at local injury area of the spinal cord and do not consider the connection between the brain and spinal cord, thus achieving a far lower effect than expected. Our previous study found that the recovery of motor function in patients with SCI is mainly dependents on the functional compensation of the brain’s M1 region, higher spontaneous nerve activity in the M1 region after SCI was associated with better recovery of motor function (Hou et al., 2016). Moreover, increasing evidence has shown that using neuromodulation techniques (such as TMS or direct current stimulation) stimulation of the M1 region has an important factor in nerve regeneration and motor function recovery after SCI (Yozbatiran et al., 2016; Cortes et al., 2017). For example, Bonizzato and his colleagues (Bonizzato et al., 2018) proved that compared with continuous spinal cord stimulation, brain stimulation accelerated and enhanced long-term recovery of motor function after SCI. Zareen et al. (2018) showed that chronic M1 electrical stimulation could reactivate different signal pathways in axon growth and synapse formation, thus cause axonal buds after corticospinal tract damage caused by SCI or stroke, ultimately promote functional recovery. The findings of this study are consistent with the results of above studies, which indicated regulating neuron activity in the M1 region of the brain may be an important link that affects motor function after SCI.

The second important finding of this study is we found that M1 glutamatergic neurons may play an important role in the motor recovery of SCI. Meanwhile, our results may also explain a potential reason that why current neuromodulation therapies are not effective enough. Firstly, the current neuromodulation therapies (electrical stimulation or magnetic stimulation) cannot accurately control the distribution of electrical/magnetic fields in brain tissue, and the activation range is not accurate. Secondly, the above methods lack of selectivity and specificity for the type of cells activated in the stimulation area. Electrical stimulation, or magnetic stimulation cannot accurately and selectively stimulate some special types of neurons in the motor cortex. These two stimulation methods activate excitatory neurons (glutamate-ergic neurons), other types of inhibitory neurons (GABA-ergic neurons), and nerve projection fibers in the stimulation area. One study confirmed that GABA receptor agonist (muscarinic alcohol) could reduce the activity of motor cortex neurons after SCI, thus cause obstruction of motor function recovery in monkeys (Nishimura et al., 2007), which indicated that activation of inhibitory neurons (GABA-ergic neurons) was not conducive to later functional recovery. Therefore, although electrical stimulation or magnetic stimulation activates excitatory neurons, it also activates inhibitory neurons. The activation range cannot be precisely limited, which may lead to the activation of excitatory and inhibitory neurons in both target and non-target areas, and eventually to inaccurate clinical efficacy. Therefore, there is an urgent need for a technique that can accurately activate excitatory neurons (glutamate-capable neurons) in the motor cortex without activating inhibitory neurons (GABA-capable neurons), increasing the excitability of vertebral cells in the motor cortex. Optogenetics can precisely activate or inhibit certain types of neurons (Ahmad et al., 2015). In this study, we used optogenetics technology and light of specific wavelengths to accurately stimulate glutamatergic neurons in M1 region of SCI rats, revealing that selective nerve stimulation can promote functional recovery after SCI. The above findings may provide a potential direction for the precise neuromodulation of SCI in the future studies.

In addition, this study also found that the mechanism of stimulating M1 glutamatergic neurons to promote functional recovery is related to the neurofilament regeneration in the injured spinal area, thus promoting repair of injured tissues. According to previous studies, the main reason for promoting recovery by electrical stimulation or magnetic stimulation was also to promote the expression of neurotrophic factors in the injured area. Geremia et al. (2007) have proved that brief stimulation can increase the expression of GAP-43 and BDNF, thus achieving the effect of motor neuron regeneration. Cheng et al. (2014) found that selective light stimulation of the primary motor cortex can promote functional recovery of stroke rats due to increased expression of activity-dependent contralesional cortical neurotrophic factors including BDNF and NGF. In this study, we also confirmed that light stimulation activation of M1 glutamatergic neurons could increase the expression of neurotrophic factors and promote axonal regeneration in the spinal injury area.

Our study has the following limitations: 1. The excitability of inhibitory neurons (GABA neurons) in the M1 area was not further inhibited by yellow light through intervention measures, and the cell types promoting functional recovery were further defined from different aspects. 2. Electrical stimulation or magnetic stimulation was not set as the control group; thus, it remained unclear whether accurate light stimulation is more effective than conventional electromagnetic stimulation in promoting SCI function recovery. 3. Not enough neurotrophic factors were detected to further clarify the recovery of SCI. However, this article is only an exploratory study on optogenetic stimulation of SCI rats. The primary purpose was to confirm the feasibility and effectiveness of this method. Future studies should identify this difference in efficacy.

To sum up, our results showed that optogenetics technology could be used to activate glutamate neurons in the M1 region accurately. At the same time, the expression of neurotrophic factors, such as NGF and BNDF in the injured spinal cord, could be significantly increased, thus promoting axonal regeneration and finally improving motor function after SCI. This indicates that accurate activation of M1 glutaminergic neurons may be a new therapeutic direction for SCI in the future. Of course, whether optogenetics technology can be applied to clinical SCI patients in the future remains to be determined through the use of gene therapy technology.

## Data Availability Statement

The raw data supporting the conclusions of this article will be made available by the authors, without undue reservation.

## Ethics Statement

The animal study was reviewed and approved by Animal Care and Use Committee of Army Medical University. Written informed consent was obtained from the owners for the participation of their animals in this study.

## Author Contributions

JH and HL designed and supervised the study. WD, GW, LM, and ZF performed the experiments. WD and HC performed the data analysis. JH, WD, HC, and GW prepared the figures and wrote the manuscript. HC, JS, MT, and HL helped for revising the manuscript. All authors contributed to the article and approved the submitted version.

## Conflict of Interest

The authors declare that the research was conducted in the absence of any commercial or financial relationships that could be construed as a potential conflict of interest.
