# Supplementary figures and images for: Optogenetic Neuronal Stimulation Promotes Functional Recovery After Spinal Cord Injury
Source: Front Neurosci. 2021 Apr 9;15:640255. doi: 10.3389/fnins.2021.640255 (PMC8062867; doi:10.3389/fnins.2021.640255)

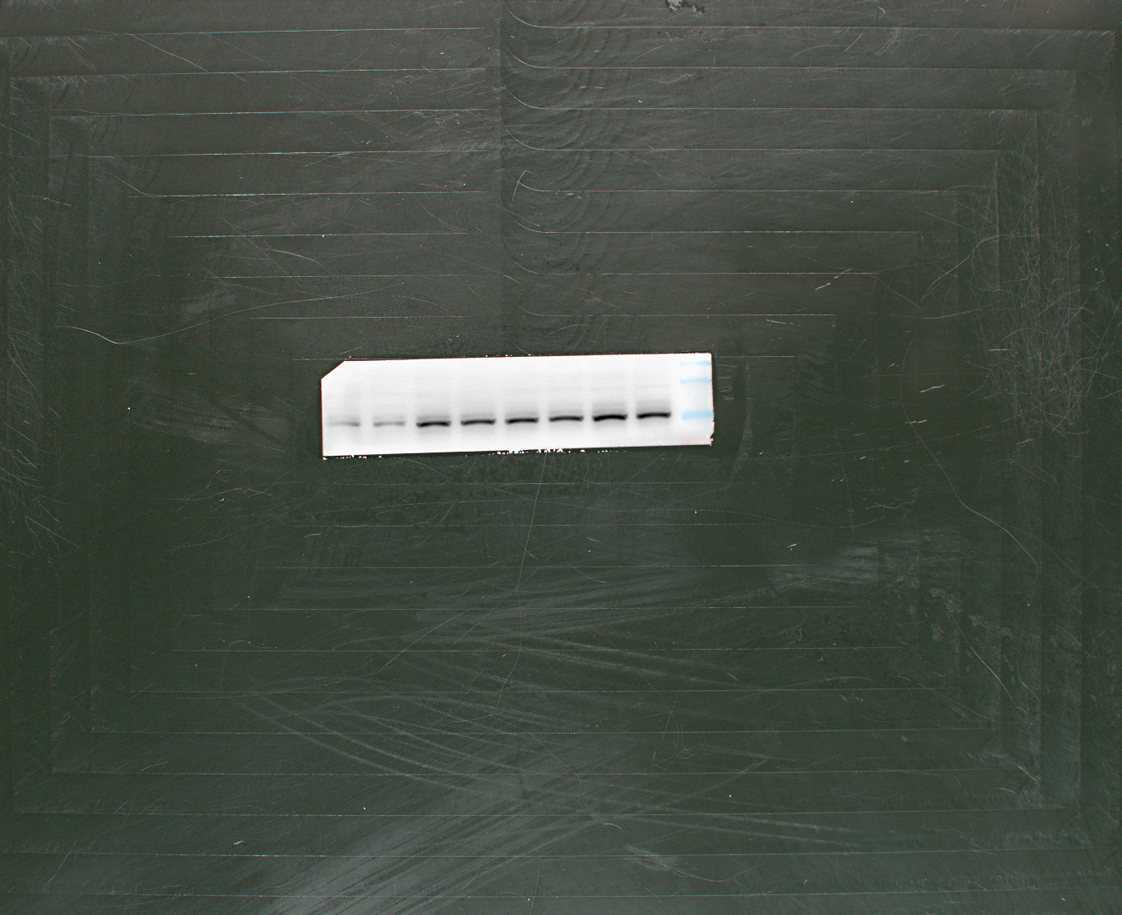

Supplement: Supplementary file 2 [file Data_Sheet_1.ZIP › original blots/BDNF.2.Tif]

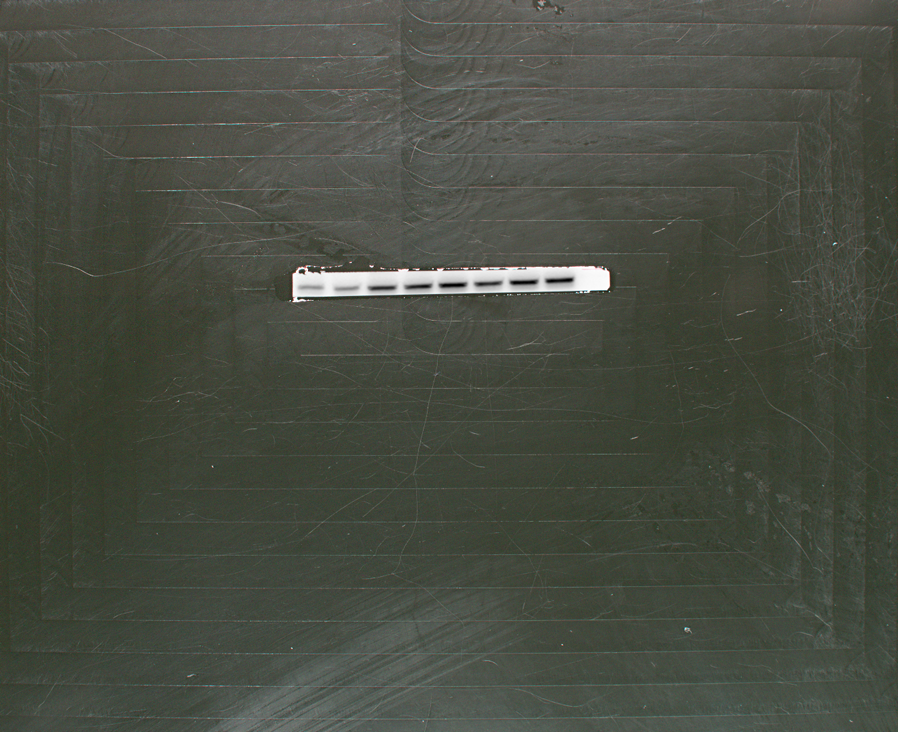

Supplement: Supplementary file 2 [file Data_Sheet_1.ZIP › original blots/GAP-43.2.Tif]

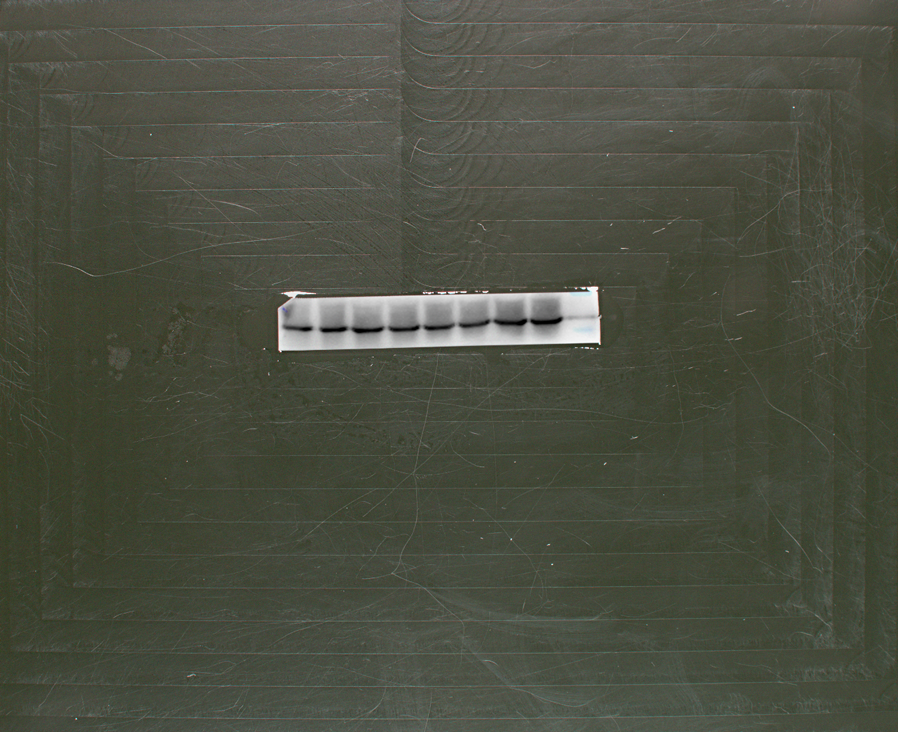

Supplement: Supplementary file 2 [file Data_Sheet_1.ZIP › original blots/NGF.2.Tif]

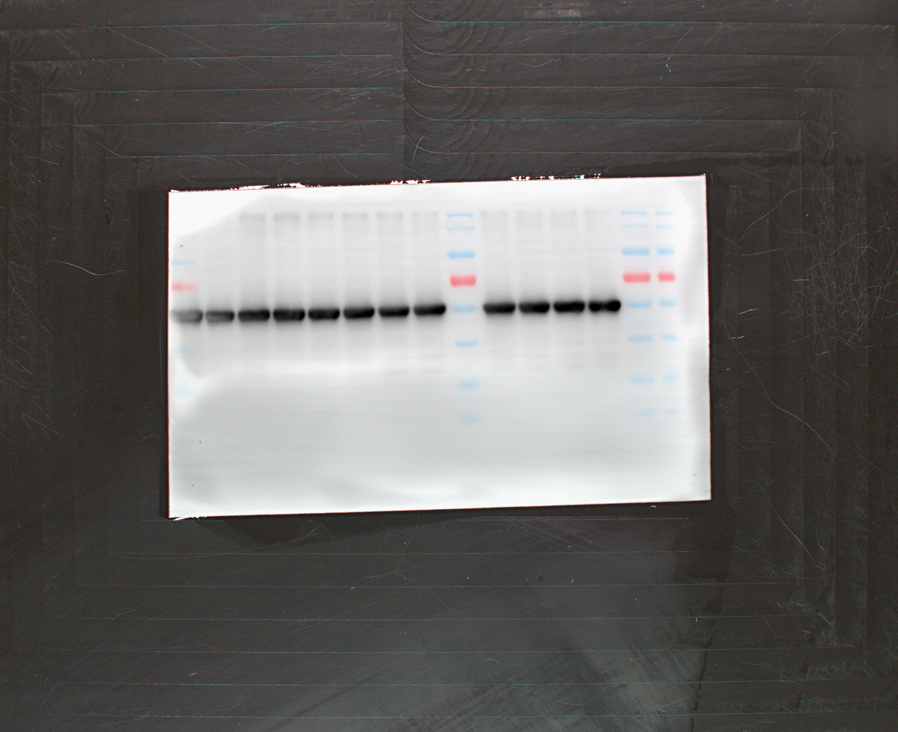

Supplement: Supplementary file 2 [file Data_Sheet_1.ZIP › original blots/TUBULIN.2.Tif]
